# The Mind and Liver Test: A New Approach to the Diagnosis of Minimal Hepatic Encephalopathy in Resource-Poor Settings

**DOI:** 10.1155/2014/475021

**Published:** 2014-12-08

**Authors:** Saurav Das, Sajjadh M. J. Ali, James Seward, Jayanthi Venkataraman

**Affiliations:** ^1^Department of Medical Gastroenterology, Stanley Medical College and Hospital, Chennai 600001, India; ^2^Banner Alzheimer's Institute, E Willetta Street, Phoenix, AZ 85006, USA; ^3^Global Health City, Perumbakkam, Chennai, Tamil Nadu 600100, India

## Abstract

*Background and Aims*. Minimal hepatic encephalopathy (MHE) is diagnosed using neuropsychometric tests or neurophysiological tests that are either inapplicable to illiterate patient population in resource-poor settings or require sophisticated and expensive equipment. The available tests assess discrete domains of mental impairment. Our aim was (a) to design a neuropsychometric test that measures all domains of mental impairment in MHE using one metric; (b) to evaluate its sensitivity, specificity, and reproducibility. *Methods*. The mind and liver test (MALT), a psychometric test assessing cognition, memory, and psychometric impairment, each on a scale of 20, was designed keeping in mind the requirements of a universal test. 40 cirrhotics and 36 controls were subjected to critical flicker frequency (CFF) and MALT in same sitting. ROC curve was plotted for MALT using CFF as gold standard. Bland-Altman plot was used to find test-retest agreement. *Results*. CFF values and MALT scores varied significantly between the cases and the controls (*P* < 0.05). MALT was 94% sensitive and 83% specific. Using ROC with CFF as gold standard, the AUC for diagnosis of MHE using MALT score was 0.89. Test-retest agreement was high (ICC = 0.89). *Conclusion*. In this pilot study, MALT proved to be highly sensitive, specific, inexpensive, and reproducible.

## 1. Introduction

Hepatic encephalopathy is a major complication of cirrhosis. It is characterized by neuropsychiatric manifestations. In minimal hepatic encephalopathy (MHE), patients have alterations in sleep pattern, and there is difficulty in performing even day-to-day routine activities like driving a vehicle, doing simple calculations, and so forth [1–3]. This is often due to an impairment in multiple faculties of the mind. Initially there is a mild impairment in cognition and psychomotor skills which then progress to a gross impairment in orientation and general consciousness. These manifestations are potentially reversible, especially, if the diagnosis is made early in the course of the disease [4, 5].

The diagnosis of minimal hepatic encephalopathy (MHE) is made using a battery of neuropsychometric or neurophysiological tests [2]. The existing neuropsychometric tests include paper-pencil tests like the number connection tests A and B, the figure connection test (FCT), trail tracing test, block design test, digit symbol test, and the comparatively recent inhibitory control test [6]. Inaccuracies in the paper-pencil tests have been widely reported [7, 8]. Also, there is a lack of consensus on the diagnostic criteria and a limited correction for educational level and age in these studies [4]. The FCT was designed for the Indian population and has been widely accepted as a sensitive test to detect MHE in illiterate individuals [9]. However, this test assesses only a few discrete domains of impairment (attention and psychomotor skills). Similarly the inhibitory control test primarily assesses attention deficit [10]. As a result, much of the tests hitherto mentioned are seldom used in day-to-day clinical practice [11]. More recent objective neurophysiological tests include critical flicker frequency (CFF) and, auditory and visual evoked potentials. All these tests involve expensive specialized equipment and trained experienced personnel [7, 11]. There is therefore a need to design an inexpensive test which would measure all the impaired domains of the mind using the same metric [12].

We designed a test that would examine the three important domains—cognition, memory, and psychomotor skills—and used this test in patients with chronic liver disease to detect MHE.

## 2. Materials and Methods

### 2.1. Design of the Mind and Liver Test (MALT)

The impaired domains of mental function in MHE have an impact on the quality of daily life [13]. The mind and liver test (MALT) was conceptualized on a scientific basis and was designed to measure the daily mental needs of an individual as a whole, and not for just one specific domain. MALT was designed such that it served as a single metric measure for all impaired domains like cognition, memory, and psychometric skills.

In order to design the specific tasks which make up the MALT, a questionnaire investigating the specific daily mental demands encountered by the target population was administered to 100 individuals attending the out-patient liver clinic of Institute of Gastroenterology and Hepatology, Stanley Hospital, Chennai, India. Except for 5 patients who were either professionals or businessmen, the rest belonged to lower socioeconomic status and were laborers, unskilled workers, or farmers (Kuppuswamy's socioeconomic status scale) [14]. Based upon the results of the questionnaire, MALT items were chosen to be relevant to these daily demands.

For* cognition* the questions were designed to identify certain animals, ability to follow the sequence of a natural phenomenon (e.g., morning-noon-evening-night), and ability to perform simple mathematical calculations needed during daily transactions (e.g., transaction at the shop). For* memory* the questions tested immediate, short term, and long term recall separately. We also tested the ability to recall a visual as well as an auditory experience. The* psycho-motor* abilities were tested by asking the patient to copy certain figures on a calibrated graph paper, to build a three-dimensional structure of a wall using building blocks, to cut along a drawn line using a scissor, and to trace along a narrow path (analogous to a line tracing test).

All the three tests were designed using a colorful board, set of printed question cards, plastic building blocks, animal models, an audio device, and an instruction* cum* scoring sheet. For convenience, the three components of neuropsychometric analysis were color coded. Red steps tested memory, green steps tested cognition, and yellow steps tested psycho-motor skills [15].

These tasks were grouped into three different sections:

 Section 1: figure the field,
 Section 2: home sweet home,
 Section 3: road not taken.

Section 1 included steps like identification of animals and vegetables on the countryside/neighborhood, and identification of well-known sequences of events like sowing seeds, transplanting the saplings, harvesting, and finally transporting to the city. In this section, we also asked the patient to build a wall using blocks. The failure to perform this task was considered a sign of psycho-motor impairment.

Section 2 included steps like remembering the arrangement of things on the shelves at home and ability to narrate the steps of a task performed at home daily, such as washing a cloth or making tea. Impairment in memory and organization reflected directly on the performance in these steps. In the same section we tested the ability of the patient to identify and remember sounds by using different stimuli, such as a church bell or an ambulance siren. Examinees were also asked to cut paper using a pair of scissors, a measure of psychomotor abilities.

Section 3 included tasks that tested the numerical abilities of the patient (like we asked the patient to perform small transactions at the shop) and tasks that asked them to identify places that they may visit on a daily basis, like a temple, a water pump, or a market place. Short term memory was tested by asking the patient to recall the short list of things to buy from the shop that was narrated to him in Section 2. We included a test analogous to the trail tracing test in Section 3 where the patient had to negotiate a narrow path from the shop back to home, using his index finger along the trail. Performance on this step can be impaired in patients with psycho-motor impairment.

**Figure 1:**
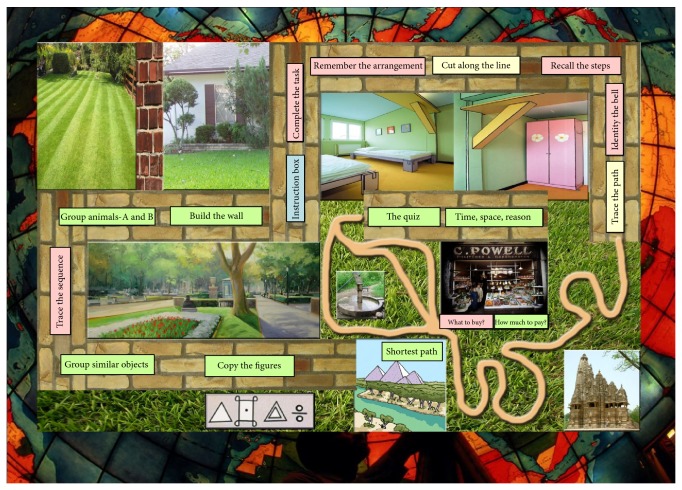
MALT test board. The test is administered clock-wise starting from Step 1, “Copy the figures.” The red steps test memory, green-cognition, and yellow-psychomotor skills. The scores are noted on instruction cum scoring sheet (Table 2).

Two other tests were performed on the MALT board, which could affect the daily activities in MHE. These included (a) divided attention: ability to observe images on the board that were not included as a part of the task; (b) judgment ability: to select the shortest of the three shown paths.  Table 1 shows the steps included in the three sections of the test that appear on the MALT board (Figure 1) and the corresponding mental function that each step evaluates.  Table 2 shows the instruction cum scoring sheet.

### 2.2. Interpretation of MALT

To score the performance on MALT, we took into account both accuracy and speed of performance. Based upon the performance of initial 10 controls in the test, we fixed an upper limit of 1 minute for each of steps  3, 5, and 6. The maximum scores on the entire test was 60 and 20 each on cognition, memory, and psychomotor skills. Higher scores implied greater impairment in mental function.

Informed consent was obtained from each patient included in the study and the study protocol conforms to the ethical guidelines of the 1975 Declaration of Helsinki as reflected in a priori approval by the ethics committee of Stanley Medical College and Hospital. The study was conducted at the Department of Medical Gastroenterology, Stanley Hospital. Forty proven cirrhotic patients were recruited as cases from among the out-patients and in-patients registered in our department. The diagnosis was based on clinical presentation and ultrasound findings. Patients with neurological deficit, obvious overt encephalopathy, alcohol abuse 72 hours prior to the test, or those on sedatives or psycho-active drugs in the preceding 24 hours were excluded. Patients with cardiac failure, renal disease, and chronic obstructive airway disease were also excluded. It was ensured that the patients had not received lactulose, lactitol, or antibiotics like rifaximin, neomycin, or quinolones in the past three months. Controls were healthy individuals screened at the Master Health Checkup scheme in the hospital.

All controls and patients had critical flicker frequency test using portable battery-powered Hepatonorm Analyzer, Accelab GmbH, Kusterdingen, Germany. The test was done in a quiet dark room. The analyzer evoked an intrafoveal light stimulus with defined pulses [7, 16]. The frequency of pulsation decreased gradually. The patient was asked to press a hand held switch when the change was apparent. The procedure was repeated 5 times to make the subject familiar with the process and 9 readings were recorded. The mean for each patient was calculated. A value below the 38 Hz mark was considered diagnostic of MHE [5, 7, 16].

The subjects (cases and controls) were then brought into a well lit room and were allowed a relaxation time of 15 minutes after which MALT was administered by two trained medical professionals (SD and SM). The patient and the tester are seated across a table with the test board, question cards, an audio device, and a table clock. The performance of the patient was noted on the instruction/scoring sheet, including time to completion.

Both MALT and CFF were done in the same sitting. Patients were randomly assigned to MALT or CFF as the first test. Both tests were executed by two different persons (SD and SM) who were blinded to the other examiner's results. The performers administering the tests were trained by experienced clinicians so as to minimize the intra- and interobserver variations.

Chi-square test was used to assess differences between qualitative variables. Student's *t*-test was used to compare the mean CFF values and mean MALT scores between the cases and the controls. Preliminary analyses provided data for receiver-operating characteristic curves to determine the optimum cut-off value for MALT score for the diagnosis of MHE. The best sensitivity and specificity was found at a cut-off score of 20. Using the CFF performance as the reference standard, statistical analysis was performed using Student's *t*-test and the receiver-operating characteristic (ROC) curve. The cirrhotics were classified as “cirrhotics with MHE” and “cirrhotics without MHE,” based upon their CFF readings. These groups were compared among themselves and with the control groups. SPSS version 10 was used for all statistical analyses and in the generation of the plots.

**Figure 2:**
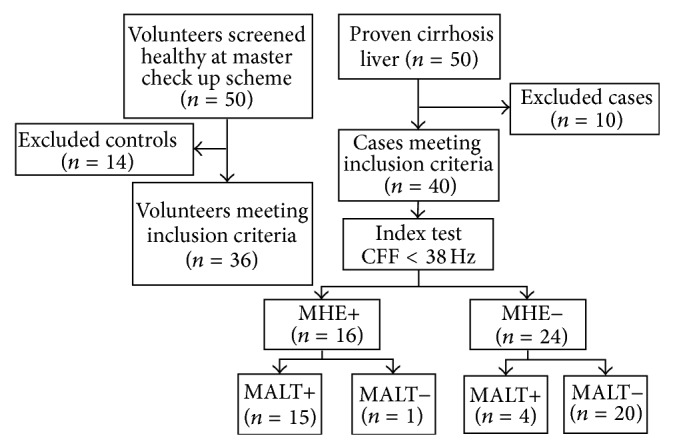
Study design flowchart according to the STARD guidelines.

The test-retest reproducibility was assessed using the Bland-Altman plot. Twenty subjects including 10 cases and 10 controls were administered MALT twice at an interval of a six hours. It was ensured that patients being evaluated were not on lactulose, lactitol, neomycin, or any sedative meanwhile. The test was administered by the same examiner. The scores of the first and the second session of MALT were compared. The report was prepared in accordance with standards for reporting diagnostic accuracy, STARD guidelines (Figure 2) [17].

## 3. Results

### 3.1. Demographic and Subject Characteristics

The study comprised of 40 patients with cirrhosis (hereinafter referred to as “cases”) and included 25 males (62.5%). There were 36 healthy controls (hereinafter referred to as “controls”) and included 16 males (44.44%). The base line characteristics of controls and cases are shown in Table 3. The mean age of the cases and controls was significantly different (*P* = 0.04). There was no difference in sex distribution (*P* = 0.18), educational status (*P* = 0.93), and monthly income (*P* = 0.09) among the cases and the controls.

The etiology of cirrhosis was alcohol in 15 patients (37.5%), HCV in 13 (32.5%), nonalcoholic fatty liver disease in 6 (17.5%), autoimmune in 1 (2.5%), and cryptogenic in 8 (20%). There were 22 patients (55%) belonging to Child-Pugh class A and 18 (45%) to class B. Among the cirrhotics, there were 18 variceal bleeders (45%) and 2 patients (5%) had coagulopathy. 4 patients (10%) had refractory ascites, 2 patients (5%) had spontaneous bacterial peritonitis, and 8 others (20%) had urosepsis.

### 3.2. Critical Flicker Frequency

**Figure 3:**
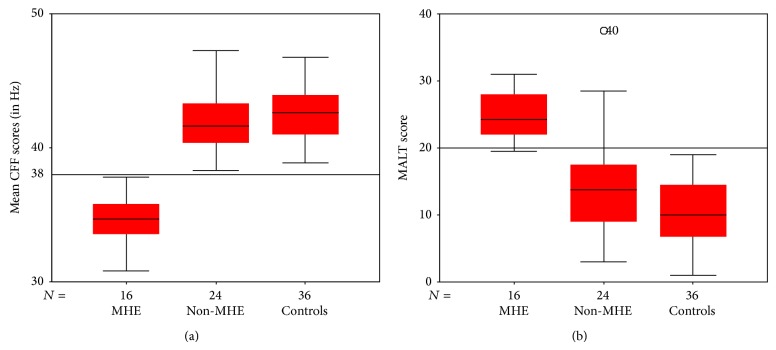
Intergroup comparison for CFF reading and MALT scores. The panel (a) compares the CFF readings between the controls, the non-MHE cirrhotics, and MHE (+) cirrhotics. The panel (b) compares the MALT scores between the same groups.

Using 38 Hz as CFF cut-off for MHE, 16 out of the 40 cirrhotics had MHE (40%), 8 each (50%) belonged to Child Class A and Child Class B. Among the 24 cirrhotics without MHE, 14 (58.33%) belonged to Child A and 10 (41.66%) to Child B at the time of MHE testing. The mean CFF for the cases was 39 ± 4.2 Hz compared to 42 ± 1.9 Hz among the controls (*P* = 0.00) (Figure 3(a)). The demographic characteristics and the lab parameters of the MHE and non-MHE groups among the cirrhotics or the “cases” are compared in Table 4.

### 3.3. MALT

**Figure 4:**
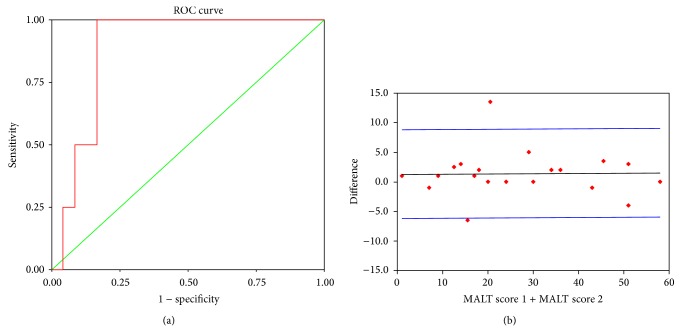
(a) ROC curve using CFF as gold standard and MALT score 20 as cut-off for the diagnosis of MHE. (b) Bland-Altman plot for the test-retest agreement of MALT.

The cases performed worse than controls on MALT. The controls scored 10 ± 5 compared to the cases, who scored 18 ± 8 (Figure 3(b)). This difference was significant (*P* = 0.00). On plotting the ROC curve (Figure 4(a)), the sensitivity and the specificity were found to be 94% and 83%, respectively, for a cut-off value set at 20. Using ROC with CFF as the gold standard, the AUC for diagnosis of MHE using MALT score was 89%. Figure 3(b) compares the mean MALT scores of MHE, non-MHE cirrhotics, and controls. MHE patients scored higher than the non-MHE and controls using the 20 mark as cut-off.

### 3.4. Test-Retest Variability

The test-retest variability was assessed using the intraclass correlation coefficient, ICC = 0.894 (*P* = 0.000). The Bland-Altman plot (Figure 4(b)) shows that the MALT retest scores of the 10 cases and 10 controls (who were subjected to the same test at an interval of 6 hours) was in agreement with the initial test scores.

### 3.5. Other Considerations

The Hepatonorm analyzer, manufactured in Germany, is an electronic device which runs on a battery that needs to be regularly changed. The price per unit is US$3053 and one day long user training costs US$230. The MALT board and its kit cost approximately US$5. The cost ratio is of the order of 10^5^. The mean time of administration for both MALT and CFF was 10 ± 2 minutes.

## 4. Discussion

The highlights of the study are (a) a significant correlation between CFF and MALT scores in the population under study (*P* < 0.05); (b) the ROC curve and the box-whisker plots indicate that MALT is a highly sensitive and specific test considering CFF as the standard; and (c) the intraclass correlation coefficient was 0.894 on assessment of test-retest variability. The sensitivity and specificity of MALT was found to be 94% and 83%, respectively, compared to earlier reported 72.4% and 77.2% for CFF when the cut-off is set at 38 Hz [18]. Though MALT may be seen to be a time taking procedure, the mean time taken for MALT and CFF did not vary significantly. The former is inexpensive test compared to CFF.

A number of neuropsychological tools have been used to diagnose cognitive deficits in patients with cirrhosis. These include an extended neuropsychological assessment, shorter batteries, and computerized tests. The extended neuropsychological assessment is based upon the expert judgment and is difficult to validate [19]. The two shorter batteries—RBANS (Repeatable Battery for Assessment of Neuropsychological Status) and PHES (Psychometric Hepatic Encephalopathy Score) are primarily paper-pencil tests. The performance on these tests is affected by the level of literacy and on the familiarity of the subject to paper and pencil among the literates. However, these tests have their own advantages. RBANS is validated on a large normative data, it is available in multiple languages and has parallel versions available for repeated testing. However, most studies are in Alzheimer's patients and the studies in cirrhotics are only few [20]. PHES was originally validated in a series of nonalcoholic patients in Germany and has normative data available from some countries. However, our knowledge of MHE has grown since those studies and, now, it is understood that an ideal diagnostic tool should reflect on those daily activities which affect the quality of life, the ability to drive, and so forth, which are often impaired in MHE [21, 22]. To overcome the shortcoming of these short batteries for the use in resource-limited settings including their dependence on the literacy level, the figure connection test (FCT) was reported by a group in India [9]. However, FCT only tests a discrete cognitive domain impaired in MHE. Computerized neuropsychological tests include cognitive drug research (CDR), inhibitory control test (ICT), and the scan test which have been validated in the UK, USA, and Italy, respectively [23–25]. We do not have the validation data for these tests in our population.

The introduction of MALT has been based on the fundamental assumption that the currently available tests for the diagnosis of MHE are designed to test for one or two domains of mental impairment, and are expensive or not widely available [10]. MALT was designed to include tasks performed on a day-to-day routine basis. In the population under study, more than 36% of the participants were illiterate and the remaining who were literate were not used to paper and pencil. This is in line with the “demise of the pencil” reported by Iduru and Mullen [22]. Moreover, age and education correction factors are absent for Psychometric Hepatic Encephalopathy Score (PHES) in our population.

The prevalence of MHE among the cirrhotics in the present study is 40%. It is in agreement with the prevalence of 41% reported in a recent study involving 200 cirrhotics from North India while on the higher side compared to 30% reported in the study from Spain using PHES [18, 26]. It would either signify that MALT included more true positives or that the prevalence is different in our population [27, 28].

MALT is a psychometric test which displays day-to-day activity on a colorful board and disagreement between administrators of the test is significantly less (85% agreement in interobserver studies). Though the tasks on the game board remain the same, the elements of the tasks are not printed on the board but on multiple cards. This is to avoid a memory effect that accompanies any psychometric test. MALT includes steps like “build the wall” and “trace the path” which are analogous to block design and trail tracing test. In MALT, the subject is not asked to connect numbers, but rather to trace the sequence of a day-to-day event from morning to night, which in true sense does not need a level of literacy.

Although the majority of surveyed hepatologists in Spain and the United States agreed that MHE was a significant problem requiring testing during out-patient visits, only a few were able to actually perform the test during their day-to-day clinical practice [29, 30]. MALT needs validation in large number of patients. In the future, when the test is available in the form of a touch screen audio-visual videogame, the test could be self-administered by the patient during the wait period in the clinic. Moreover, MALT is in the form of a colorful board. It might be interesting to note if administration of such a test has any effect on the mood and affect of the depressed cirrhotic. It will also be of interest to follow up MALT scores of patients on therapy like lactulose for hepatic encephalopathy. Also, to see a head to head analysis of MALT compared to other psychometric tests in future. However, these were beyond the scope of the present study. The choice of CFF as the gold standard in the current study was also to circumvent many problems known to be encountered with administering multiple psychometric tests [2, 31].

This study largely aimed to design a psychometric battery to diagnose MHE in our population, which is useful for the illiterate patients and to check its reliability. The high sensitivity and specificity of MALT, its ease of administration, reproducibility, and cost-effectiveness seen in this pilot beckon further validation studies involving larger sample sizes.

## Supplementary Material

MALT is a psychometric test which displays day-to-day activity on a colorful board. Though the tasks on the game board remain the same, the elements of the tasks are not printed on the board but on multiple cards. This is to avoid a memory effect that accompanies any psychometric test. A few such cards are provided in the supplementary material.

## Figures and Tables

**Table 1:** MALT-steps summary.

Section	Task	Corresponding mental function
(1) Figure the field	Copy the figures	Visuospatial abilities
	Group similar objects	Recognition
	Trace the sequence	Executive function
	Name the animals	Naming
	Build the wall	Constructional ability

(2) Home sweet home	Complete the task	Executive function
	Remember the arrangement	Immediate visual memory
	Cut along the line	Psychomotor skills
	Recall the steps	Attention
	Identify the sound	Auditory recall

(3) The road not taken	Trace the path	Psycho-motor skills
	What from shop?	Delayed visual recall
	How much to pay?	Calculation
	Choose the shortest path.	Decision making
	Quiz	Divided attention
	Time, space, and reason	Abstraction∣Orientation

**Table 2:** MALT instruction cum scoring sheet.

Sl	Method of administering the test	Instruction to fill up the next column	Step score	Final count
1	Copy the figures The subject is asked to draw the shown geometrical figures (printed on the board) onto a provided ruled paper, without altering the size (The original figures cross three units on the same paper)	Symmetrical figure…0 else…1 Number of units crossed	*⋯* 3-…	*⋯*

2	Group similar objects The subject is shown one card (of a set of cards) showing the pictures of a few things belonging to two different categories (birds and vegetables, say). The subject is asked to place the blocks of the same color on objects of the same type	Number of objects misgrouped	…/2	*⋯*

3	Trace the sequence The subject is shown a card to trace a well known sequence (like seed-sapling-tree-wood log-table) The examiner waits for a maximum of 1 minute, else proceeds to the next step	Score the time in seconds 0–10 s…0, 11–20 s…1, 21–30 s…2, 31–40 s…3, >40 s…4	*⋯*	*⋯*

4	Name the animals A: the subject is shown an animal (a cow, say) and is asked to name it	Score time in seconds <5 s…0, 5–15 s…1, >15 s…2	*⋯*	*⋯*
	B: the subject is asked to separate animals of a certain kind from among the toys	<15 s…0, else 1	*⋯*	

5	Build the wall The subject is asked to build a two layered wall on the given line using the provided blocks The examiner waits for a maximum of 1 minute and then proceeds to the next step	A well built wall…0 Asymmetrical…1 Unable to build…2 (Asymmetry is defined as inability to place the blocks linearly either along the line in the game board or inability to stack two layers one over the other)	*⋯*	*⋯*
	Instruction box The subject is made to hear three different bell sounds like the alarm, ambulance, and church bell using the audio device. He is also asked to be vigilant about the things he sees in the rest of the game			

6	Complete the task The patient is asked to arrange in sequence, the cards depicting the steps of a daily act performed by people at home The examiner waits for a maximum of 1 minute and then proceeds to the next step	Able to complete the task…0 Else…1	*⋯*	*⋯*

7	Remember the arrangement The subject is shown an arrangement in the shelf (image available on a question card) and it is narrated once for convenience Immediately following this, the subject is asked to recall the arrangement	Number of objects recalled correctly	(6-…)/2	*⋯*

8	Cut along the line The subject is asked to cut along a line with both indentation and curve The graph sheet on the reverse is analyzed for deviation	Deviation (in small units) 5–10…3 >10…5	*⋯*	*⋯*
		Time (in seconds) >45…5, else…0	*⋯*	

9	Recall the steps of the game till this point, in no specific order	Number of steps recalled	8-…	*⋯*
	The examiner asks a question pertaining to the sequence of the steps (what comes after X, say)	Correct answer…0 Else…1	*⋯*	

10	Identify the sound of the bell One of the three bells sounded in the instruction box is sounded again	Unable to identify…1 Else…0	*⋯*	*⋯*

11	Trace the path Subject is asked to trace a 5 mm thick path to the shop	Time (in seconds) >30 s…5, 16–30 s…3 Else…0	*⋯*	*⋯*
		Deviation (in small grids on the graph paper) <5…0, 5–15…3, 16–25…4, >25…5	*⋯*	

12	What from the shop? The subject is asked if he remembers what he had to get for the empty basket on the lowest shelf of the rack (This tests the recall of the question card shown in Step 7)	Incorrect…1 Else…0	*⋯*	*⋯*

13	How much to pay? The subject is given a simple calculation pertaining to day to day transactions	Incorrect…1 Else…0	*⋯*	*⋯*

14	Choose the shortest path The subject is asked to choose the shortest path back home	Incorrect…1 Else…0	*⋯*	*⋯*

15	Quiz The subject is asked if he saw certain things on the game board while tracing the path to the shop	Number of questions answered	4-…	*⋯*

16	Time, space, and reason	A wrong answer to the question pertaining to spatial and temporal orientation, and silence in response to the reasoning question would be marked as “D” (for disoriented)		

**Table 3:** Demographic characteristics of cases and controls.

Demographic characteristic (Mean ± SD)	Controls (*n* = 36)	Cases (*n* = 40)
Age (years)	42 ± 9.7^*^	46 ± 8.3^*^
Male : female	16/20	25/15
Education (not educated/educated)	13/23	13/27
Income per month (in US dollars)	15.36 ± 14.51	10.77 ± 7.40

^*^ *P* < 0.05.

**Table 4:** Demographics within the cirrhosis group.

	Cirrhotics without MHE (CFF−) (*n* = 24)	Cirrhotics with MHE (CFF+) (*n* = 16)
Age	43.5 ± 8.1^*^	50 ± 7.2^*^
Years of education	6.4 ± 4.5^*^	3.06 ± 3.7^*^
Gender (M/F)	16/8	9/7
Cirrhosis etiology (Alcohol/HCV/HCV+Alcohol/NASH/Others)	09/05/00/04/06	03/05/03/02/03
MELD score (median)	11	14
Child score (A/B)	14/10	8/8
Total bilirubin (median)	1.5	1.6
INR (median)	1.1^*^	1.3^*^
Serum creatinine (median, in mg/dL)	0.73^*^	0.86^*^
MALT scores	14.8 ± 7.8^†^	24.8 ± 3.5^†^

^*^ *P* < 0.05 between two groups.

^†^ *P* < 0.01 between two groups.
