# Supplementary figures and images for: The Mind and Liver Test: A New Approach to the Diagnosis of Minimal Hepatic Encephalopathy in Resource-Poor Settings
Source: Int J Hepatol. 2014 Dec 8;2014:475021. doi: 10.1155/2014/475021 (PMC4274711; doi:10.1155/2014/475021)

Supplementary Material-

Fig. 1 : Question cards


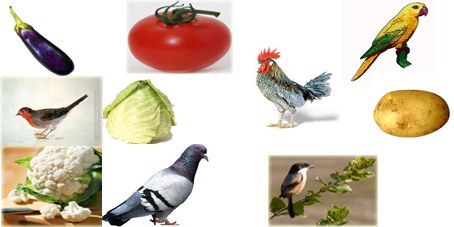


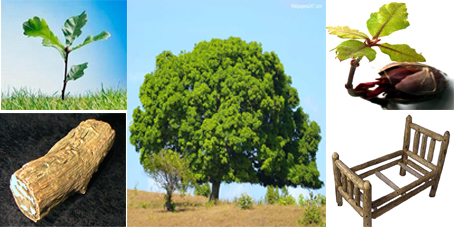

Supplement: Supplementary file 1 — MALT is a psychometric test which displays day-to-day activity on a colorful board. Though the tasks on the game board remain the same, the elements of the tasks are not printed on the board but on multiple cards. This is to avoid a memory effect that accompanies any psychometric test. A few such cards are provided in the supplementary material. [file 475021.f1.docx]
